# Spatial and temporal trends in social vulnerability and COVID-19 incidence and death rates in the United States

**DOI:** 10.1371/journal.pone.0248702

**Published:** 2021-03-24

**Authors:** Brian Neelon, Fedelis Mutiso, Noel T. Mueller, John L. Pearce, Sara E. Benjamin-Neelon

**Affiliations:** 1 Division of Biostatistics, Department of Public Health Sciences, Medical University of South Carolina, Charleston, South Carolina, United States of America; 2 Charleston Health Equity and Rural Outreach Innovation Center (HEROIC), Ralph H. Johnson VA Medical Center, Charleston, South Carolina, United States of America; 3 Department of Epidemiology, Johns Hopkins Bloomberg School of Public Health, Baltimore, Maryland, United States of America; 4 Welch Center for Prevention, Epidemiology and Clinical Research, Johns Hopkins University, Baltimore, Maryland, United States of America; 5 Division of Environmental Health, Department of Public Health Sciences, Medical University of South Carolina, Charleston, South Carolina, United States of America; 6 Department of Health, Behavior and Society, Johns Hopkins Bloomberg School of Public Health, Baltimore, Maryland, United States of America; Florida State University College of Medicine, UNITED STATES

## Abstract

**Background:**

Socially vulnerable communities may be at higher risk for COVID-19 outbreaks in the US. However, no prior studies examined temporal trends and differential effects of social vulnerability on COVID-19 incidence and death rates. Therefore, we examined temporal trends among counties with high and low social vulnerability to quantify disparities in trends over time.

**Methods:**

We conducted a longitudinal analysis examining COVID-19 incidence and death rates from March 15 to December 31, 2020, for each US county using data from USAFacts. We classified counties using the Social Vulnerability Index (SVI), a percentile-based measure from the Centers for Disease Control and Prevention, with higher values indicating more vulnerability. Using a Bayesian hierarchical negative binomial model, we estimated daily risk ratios (RRs) comparing counties in the first (lower) and fourth (upper) SVI quartiles, adjusting for rurality, percentage in poor or fair health, percentage female, percentage of smokers, county average daily fine particulate matter (PM_2.5_), percentage of primary care physicians per 100,000 residents, daily temperature and precipitation, and proportion tested for COVID-19.

**Results:**

At the outset of the pandemic, the most vulnerable counties had, on average, fewer cases per 100,000 than least vulnerable SVI quartile. However, on March 28, we observed a crossover effect in which the most vulnerable counties experienced higher COVID-19 incidence rates compared to the least vulnerable counties (RR = 1.05, 95% PI: 0.98, 1.12). Vulnerable counties had higher death rates starting on May 21 (RR = 1.08, 95% PI: 1.00,1.16). However, by October, this trend reversed and the most vulnerable counties had lower death rates compared to least vulnerable counties.

**Conclusions:**

The impact of COVID-19 is not static but can migrate from less vulnerable counties to more vulnerable counties and back again over time.

## Introduction

Severe acute respiratory syndrome coronavirus 2 (SARS-CoV-2), the cause of coronavirus disease 2019 (COVID-19), has created a global public health crisis since its onset in late 2019. As of late February 2021, there have been over 27 million confirmed COVID-19 cases and nearly a half a million deaths in the United States (US) alone [1]. Overwhelming evidence has found that the pandemic has disproportionately affected people of color, older individuals, and those of lower socioeconomic status [2–10]. Several studies have shown that African Americans have higher incidence and death rates than non-Hispanic whites [6, 8, 9, 11]. Two studies also reported that COVID-19 infection rates are greater in US counties and in states with high Latinx populations and monolingual Spanish speakers [4, 7]. Further, older age has been associated with an increased risk of death among those infected with COVID-19 [5, 12, 13]. Underlying health conditions and comorbidities may partially explain these associations [5], but do not fully account for the disproportionate burden. Recent studies suggest that social determinants of health and community contextual factors contribute to these disparities, and that socially vulnerable communities are at highest risk for COVID-19 outbreaks [6, 14–16].

Protecting vulnerable populations is critically important during the COVID-19 pandemic, as these groups are generally at higher risk for adverse health outcomes [17, 18]. Hurst et al. define vulnerability as an identifiably elevated risk of incurring greater wrong or harm [19]. One type of vulnerability–social vulnerability–has been used by the Centers for Disease Control and Prevention (CDC) to identify communities most at risk when faced with adverse events that may impact health, such as natural disasters or disease outbreaks. The CDC developed the social vulnerability index (SVI) to assist federal, state, and local governments in targeting and mobilizing resources for at-risk counties in response to adverse events.

Recent studies have demonstrated the importance of considering social vulnerability in both COVID-19 cases and deaths, although the findings have been somewhat inconsistent [20–23]. Karaye et al. examined associations between the SVI and cumulative COVID-19 cases on May 12, 2020 [20]. They found that SVI total score was associated with increased rates of COVID-19. However, the authors found no association when they examined six states with high testing rates. Khazanchi and colleagues conducted an analysis of COVID-19 cases and deaths through April 19, 2020, and found that those living in the most vulnerable counties (highest SVI) had greater risk of infection and death [22]. Nayak et al. examined associations between the SVI and COVID-19 incidence and case fatalities through April 4, 2020, and found a significant association between social vulnerability and case fatality but not incident cases [21]. Finally, Wang and colleagues found that social vulnerability and COVID-19 incident cases and deaths had spatially varying associations; however, the authors did not adjust for potential confounders [23]. Notably, all studies were cross-sectional and conducted at different time points early in the pandemic, which might contribute to the inconsistent findings. In fact, to date, no prior studies have examined longitudinal trends in social vulnerability and COVID-19 incidence and death rates in an effort to determine how these relationships change over time. Therefore, the purpose of this study was to examine temporal trends among counties with high and low social vulnerability and to quantify disparities in these trends over time.

## Materials and methods

### Overview

We conducted a retrospective longitudinal analysis examining COVID-19 incidence and death rates from March 15, 2020 to December 31, 2020 for each of the 3,142 US county and county equivalents based on their unique Federal Information Processing Series (FIPS) codes [24, 25]. Specifically, we modeled the temporal trend in daily incidence and death rates for each county and assessed differential risks by county-level social vulnerability. We hypothesized that highly vulnerable counties would have higher incidence and death rates compared to less vulnerable counties and that this disparity would widen over time. The Institutional Review Boards at the Medical University of South Carolina and Johns Hopkins Bloomberg School of Public Health deemed this research exempt from review.

### COVID-19 incident cases and deaths

We obtained daily COVID-19 incident case and death data from USAFacts [26] and the Johns Hopkins Center for Systems Science and Engineering [27]. Because national data are only available at the county level, we used county as the geographic unit for our analyses. Moreover, because Johns Hopkins aggregates data for some counties (e.g., the five boroughs of New York) [28], we opted to use the USAFacts data in our primary analysis, and conducted a sensitivity analysis using Johns Hopkins data. For both data sources, we downloaded daily incident case and death counts from March 15 to December 31, 2020. We obtained county population data from the 2019 population datafile compiled by the US Census Bureau [29]. We present data sources for all variables in Table 1.

**Table 1:** Data sources.

County-Level Variable	Year	Source
COVID-19 cases	2020	Johns Hopkins Center for Systems Science and Engineering [27]; USAFacts [26]
COVID-19 deaths	2020	Johns Hopkins Center for Systems Science and Engineering [20]; USAFacts [26]
Daily PCR Testing	2020	US Department of Health and Human Services
Social Vulnerability Index	2018	Centers for Disease Control and Prevention [30]
Population Size	2019	Centers for Disease Control and Prevention [31]
Population Density	2018–2019	Centers for Disease Control and Prevention [30]
Race	2019	US Census Bureau [29]
Ethnicity	2019	US Census Bureau [29]
Gender	2019	US Census Bureau [29]
Age	2019	US Census Bureau [29]
Rurality	2019	Robert Wood Johnson Foundation [32]
Poverty	2018–2019	Robert Wood Johnson Foundation [32]
Primary Care Physicians	2020	Robert Wood Johnson Foundation [32]
Smoking	2020	Robert Wood Johnson Foundation [32]
Poor or Fair Health	2020	Robert Wood Johnson Foundation [32]
Temperature	2020	National Oceanic and Atmospheric Administration [33]
Precipitation	2020	National Oceanic and Atmospheric Administration [33]
PM_2.5_	2019	Robert Wood Johnson Foundation [32]

### Social vulnerability index

We used publicly available data from the CDC’s Agency for Toxic Substances and Disease Registry to classify counties using SVI [30]. The SVI is a percentile-based measure of social vulnerability, or the resilience of communities to address stressors to health related to external hazards (e.g., natural disasters or disease outbreaks) [34]. The Geospatial Research, Analysis & Services Program within the Agency for Toxic Substances and Disease Registry created the SVI database to help public health officials identify communities that will most likely need support and resources during and after a hazardous event like a pandemic [30]. The overall index and each theme are scored from 0 to 1, with higher scores indicating greater vulnerability [30, 34]. The index was constructed using data from 15 variables from the US Census Bureau. A percentile rank was calculated for each of these variables and grouped among four themes of SVI that measure various aspects of vulnerability–these include Socioeconomic Status, Household Composition, Race/Ethnicity/Language, and Housing/Transportation [30, 34].

The Socioeconomic Status theme is composed of percentile rank data for the following variables: percentage below poverty, percentage unemployed, per capita income, and percentage with no high school diploma. For Household Composition, the variables include percentage age 65 years and older, percentage age 17 years or younger, percentage age 5 years or older with a disability, and percentage of single-parent households. The Race/Ethnicity/Language theme encompasses percentage minority and percentage who speaks English “less than well”. Finally, the Housing/Transportation theme includes data for the percentage of multiunit structures, percentage of mobile homes, percentage crowding, percentage having no vehicle, and percentage of group quarters.

For our analyses, we downloaded the 2018 county-level SVI data (the most recent available) for all 3,142 counties. One county was missing SVI data; for this county, we imputed SVI data using the national average.

### Adjustment variables

We adjusted for several variables that could help explain the differential impact of COVID-19 on upper and lower SVI counties. These variables were chosen *a priori* based on previously reported associations with COVID-19 incidence and deaths [20–22, 35–38]. As noted above, we provide data sources for all variables in Table 1. These included the percentage of each county designated as rural, the percentage of female residents, the percentage of adult smokers in the county, the number of primary care physicians per 100,000 in each county, average daily temperature (degrees Fahrenheit), and average daily precipitation (inches). We also controlled for the percentage of residents in poor or fair health, a validated measure of the overall health and comorbidity burden of the population [32]. We additionally controlled for population density, defined as the number of residents per square mile [33] and the average particulate matter of diameter ≤ 2.5 micrometers (PM_2.5_) [32], a measure of fine particulate air pollution that can compromise respiratory function. Finally, we controlled for the daily proportion of COVID-19 Viral (RT-PCR) tests performed in each state (county-level data are not currently available). We converted the number tested to a proportion by dividing the number of tests by the state population size, obtained from the US Census Bureau’s population estimate dataset [39].

Each of the above variables was included in the models examining the impact of overall SVI on cases and deaths as well as the SVI themes. However, because each SVI theme includes only the SVI variables specific to that theme, we included additional adjustment variables in the analyses involving specific SVI themes. For the Socioeconomic Status theme, we included the percentage of county residents aged 65 years and older, the percentage of non-Hispanic Black residents, and the percentage of Hispanic residents. For the Household Composition theme, we adjusted for the percentage below the federal poverty line as well as percentage of NHB and Hispanic residents; we did not adjust for age, as this is included as part of the theme. For Race/Ethnicity/Language, we adjusted for age ≥ 65 and over and percent poverty. For the Housing/Transportation theme, we adjusted for age ≥ 65, percent poverty, percent NHB and percent Hispanic. Finally, as a sensitivity analysis, we fit unadjusted models for overall SVI and the four themes.

### Statistical analysis

We fit Bayesian hierarchical negative binomial models with daily incident cases and daily deaths for each county as the outcomes. The models included penalized cubic Bsplines for both the fixed and random (i.e., county-specific) temporal effects, with knots placed approximately every two weeks over the study period (20 knots total). The models also included county population as an offset on the log scale to convert the case and death counts to population-adjusted rates. The models further adjusted for the variables described above.

To avoid overfitting the temporal splines, we assigned ridging priors to the fixed and county-specific spline coefficients–i.e., independent, mean-zero normal distributions with shared inverse gamma variances [40]. We assigned weakly informative normal priors to the corresponding regression parameters. We assigned a gamma prior to the negative binomial dispersion parameter. We developed an efficient data-augmented Gibbs sampler to aid posterior computation [41, 42]. For both the incidence case and death rate models, we ran the Gibbs sampler for 2,500 iterations with a burn-in 500 to ensure convergence.

To report results, we compared counties in the top or upper SVI quartile (most vulnerable) to those in bottom or lower SVI quartile (least vulnerable). For both quartiles, we graphed the posterior mean incidence and death rate trends for the reference covariate group along with the corresponding 95% posterior intervals (PIs). We also reported adjusted risk ratios (RRs) and 95% PIs comparing the upper and lower quartiles on each day for the overall SVI and its themes.

As sensitivity analyses, we ran unadjusted models as well as models based on Johns Hopkins case and death data. We conducted all analyses using R software version 3.6 (R Core Team 2019, R: A language and environment for statistical computing, R Foundation for Statistical Computing, Vienna, Austria).

## Results

The final analytic sample comprised 917,464 observations (3,142 counties x 292 study days). There were 786 counties in each of the upper and lower SVI quartiles. Table 2 presents county demographics overall and by upper and lower SVI quartile. The two quartiles were similar with respect to gender, age, smoking, and the total number of RT-PCR tests performed during the study period. The quartiles differed with respect to percent poverty, race/ethnicity composition and temperature, with more vulnerable counties having higher average daily temperature, suggesting that many of these counties are located in the southern US.

**Table 2:** County-level characteristics, overall and by lower and upper quartile SVI.

	Total Sample (n = 3142)	Lower Quartile SVI (n = 786)	Upper Quartile SVI (n = 786)
	Median (IQR)	Median (IQR)	Median (IQR)
% Female^a^	0.50 (0.49, 0.51)	0.50 (0.49, 0.51)	0.51 (0.49, 0.52)
% Rural^a^	0.60 (0.33, 0.88)	0.66 (0.38, 1.00)	0.59 (0.38, 0.79)
% Below poverty level^b^	13.40 (10.4, 17.5)	9.35 (7.50, 11.20)	19.90 (16.90, 23.80)
% Adult smoking^a^	0.17 (0.15, 0.20)	0.15 (0.14, 0.16)	0.20 (0.17, 0.22)
% Age 65 years and over^c^	0.19 (0.16, 0.22)	0.20 (0.17, 0.23)	0.18 (0.15, 0.20)
Race, % Non-Hispanic Black^d^	0.02 (0.01, 0.10)	0.01 (0.00, 0.02)	0.13 (0.02, 0.35)
Ethnicity, % Hispanic^d^	0.03 (0.02, 0.10)	0.03 (0.02, 0.06)	0.06 (0.03, 0.20)
Average PM_2.5_^a^	9.40 (7.70, 10.40)	8.40 (6.50, 9.90)	9.80 (8.80, 10.4)
# Primary care physicians^a^	0.00 (0.00, 0.00)	0.00 (0.00, 0.00)	0.00 (0.00, 0.00)
Population density^a^	44.80 (16.50, 119.00)	33.20 (7.39, 132.00)	38.70 (19.60, 80.70)
Average temperature (Fahrenheit)^a^^,^^e^	58.00 (44.60, 71.10)	51.50 (38.20, 66.80)	64.20 (52.50, 75.20)
Average precipitation (inches)^a^^,^^e^	3.14 (1.52, 4.95)	2.37 (1.02, 3.92)	3.80 (1.91, 5.81)

IQRs–Interquartile Range; PCR–Polymerase Chain Reaction; PM–Particulate Matter; SVI–Social Vulnerability Index

^a^ Variable included in all adjusted models

^b^ Age ≥ 65 was included only in the models for Socioeconomic Status, Race/Ethnicity/Language, and Housing/Transportation themes

^c^ Percent poverty variable was included only in the models for Household Composition, Race/Ethnicity/Language, and Housing/Transportation themes

^d^ Race variables was included only in models for Socioeconomic Status, Household Composition, and Housing/Transportation themes

^e^ Daily temperature and precipitation data averaged over entire year

### Overall SVI

**Fig 1:**
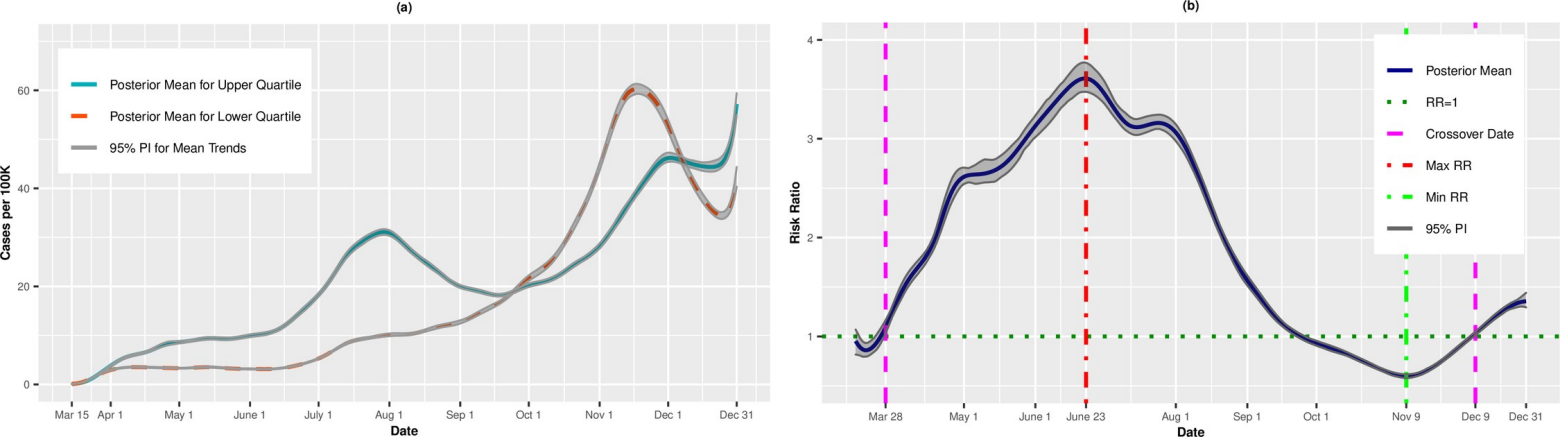
Incidence rates (A) and adjusted risk ratios (B) for upper (most vulnerable) versus lower (least vulnerable) quartiles of overall SVI.

Fig 1A presents the per capita incidence trends (expressed as cases per 100,000) for the upper (most vulnerable) and lower (least vulnerable) quartiles of SVI from the unadjusted analysis. For counties in the upper quartile, the average incidence increased steadily from March 15 (0.10 cases per 100,000; 95% PI: 0.09, 0.12) to May 12 (9.36 cases per 100,000; 95% PI: 9.08, 9.61). The incidence leveled off in mid-May before a precipitous increase through July 30 (31.10 cases per 100,000; 95% PI: 30.52, 31.68). The incident cases receded in late summer and early fall, before a final uptick from mid-September to December 31 (57.21 cases per 100,000; 95% PI: 54.94, 59.43). By comparison, the least vulnerable quartile exhibited a gradual increase in incidence through the summer and fall, achieving a maximum incidence rate of 60.23 cases per 100,000 on November 17 (95% PI: 59.25, 61.32) before tapering in late November and early December.

Fig 1B presents the posterior mean adjusted RRs comparing the upper and lower quartiles on each day. On March 15, the RR for incident cases was 0.95 (95% PI: 0.82, 1.07), suggesting that most vulnerable counties had, on average, fewer cases per 100,000 than less vulnerable \counties, although this result did not statistically differ from 1.00. In fact, through most of March, the RRs were <1.00. However, on March 28, we observed a “crossover effect” in which the RR became significantly greater than 1.00, indicating that the more vulnerable counties had higher COVID-19 incidence on average compared to less vulnerable counties (March 28 RR = 1.09, 95% PI: 1.03, 1.17). The RRs increased steadily thereafter and achieved a maximum RR of 3.61 (95% PI: 3.47, 3.77) on June 23, then decreased steadily through mid-November. Notably, on September 27, we observed a second crossover date, in which the lower (least vulnerable) quartile exhibited higher incidence compared to the upper quartile (RR = 0.97, 95% PI: 0.94, 0.99). The minimum RR of 0.60 (95% PI: 0.58, 0.62) occurred on November 9, indicating that the upper quartile had 40% lower risk of infection. However, on December 9, there was a final crossover, and the upper (most vulnerable) quartile again had higher incidence (RR = 1.03, 95% PI: 1.01, 1.05). Thus, the association between SVI and COVID-19 was not unidirectional over time, but instead exhibited an alternating pattern throughout the course of the study.

**Fig 2:**
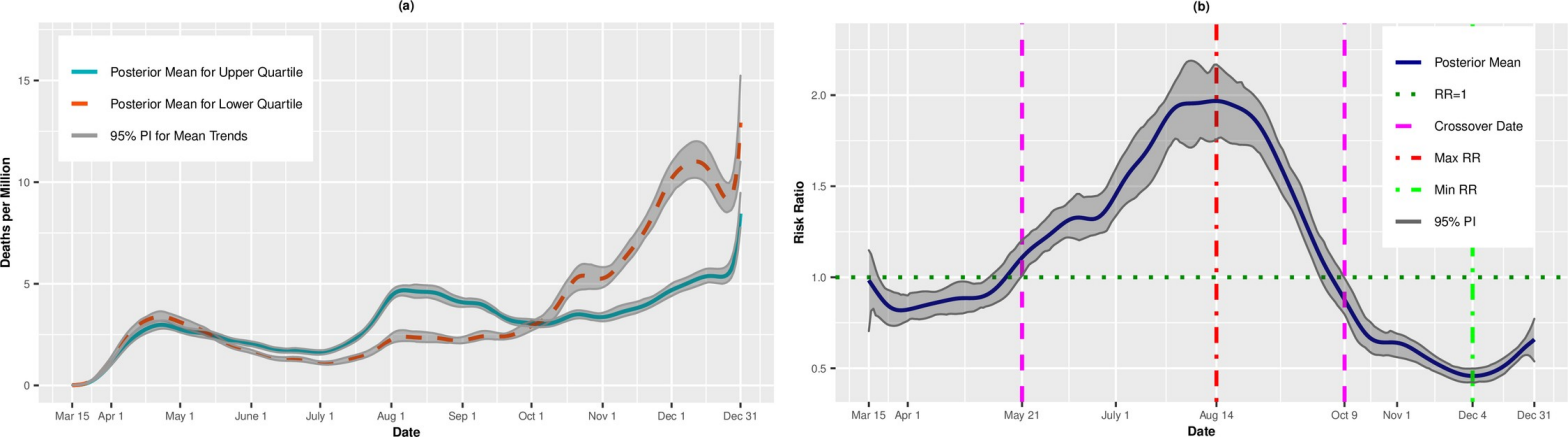
Death rates (A) and adjusted risk ratios (B) for upper (most vulnerable) versus lower (least vulnerable) quartiles of overall SVI. Maximum and minimum values, as well as the first and last crossover dates, are highlighted by vertical lines.

Fig 2A presents per capita death trends (expressed as deaths per million) for the upper and lower quartiles of overall SVI. The death rates for both quartiles increased until mid-April before leveling off through the end of June. Beginning in early July, however, the mean death rate for the upper, most vulnerable quartile increased steadily until August 7 (4.67 deaths per million, 95% PI: 4.42, 4.92). The trend for the upper quartile leveled off in early autumn before a final upswing through December 31 (8.51 deaths per million; 95% PI: 7.79, 9.43). The daily death rates for the lower (least vulnerable) quartile hovered between 2 and 3 deaths per million for most of the summer. However, beginning in late September, there was a rapid uptick in the death rate, and by December 31, there was an estimated 13.07 deaths per million (95% PI: 10.82, 15.71) on average in these least vulnerable counties. Fig 2B presents the daily RRs comparing the upper and lower quartiles. From mid-March to mid-May, the upper, most vulnerable quartile had lower death rates than the lower quartile. However, on May 21, the trend reversed, and the upper quartile had higher death rates compared to the lower quartile (RR = 1.08, 95% PI: 1.00,1.16). The RRs increased until achieving a maximum value on August 14 (RR = 1.97, 95% PI: 1.76, 2.17). On October 9, however, we observed a second crossover in which the most vulnerable counties had, on average, lower death rates than the least vulnerable counties. On December 4, the RR comparing the upper to lower quartiles was 0.46 (95% PI: 0.42, 0.50), indicating that the most vulnerable counties had on average 54% lower risk of death compared to the most vulnerable counties.

### SVI theme: Socioeconomic status

**Fig 3:**
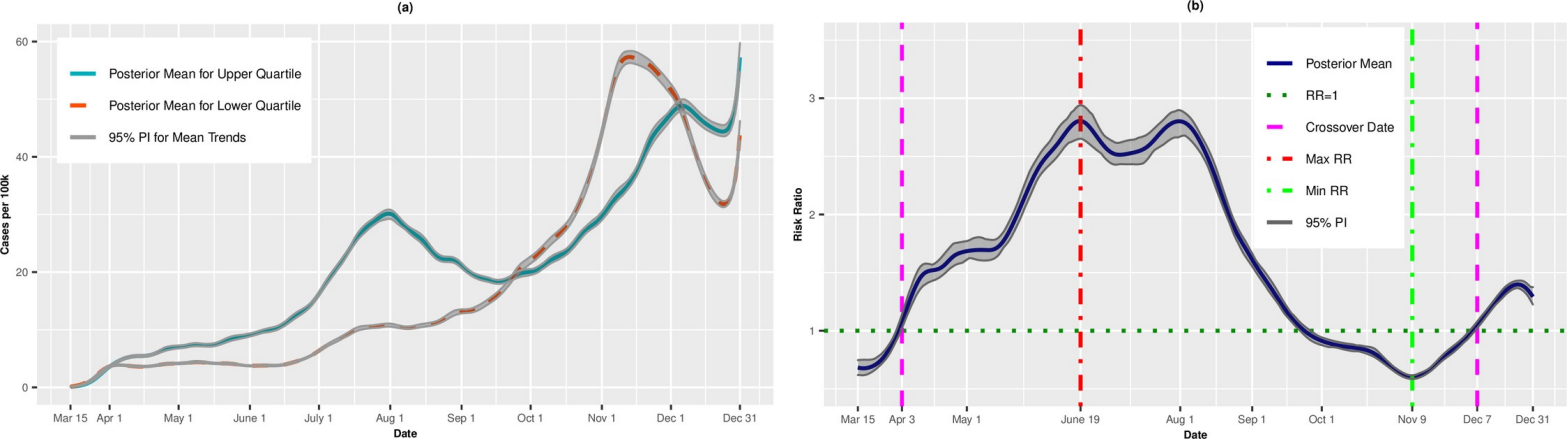
Incidence rates (A) and adjusted risk ratios (B) for upper (most vulnerable) versus lower (least vulnerable) quartiles of Socioeconomic Status.

**Fig 4:**
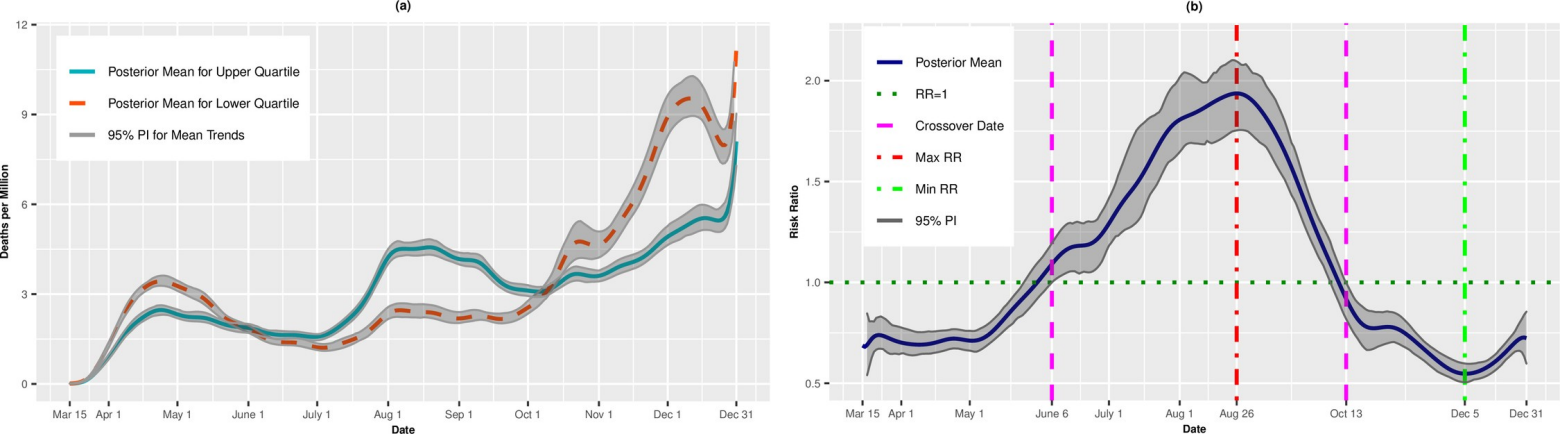
Death rates (A) and adjusted risk ratios (B) for upper (most vulnerable) versus lower (least vulnerable) quartiles of Socioeconomic Status. Maximum and minimum values, as well as the first and last crossover dates, are highlighted by vertical lines.

Figs 3A, 3B, 4A and 4B present the temporal trends and RRs for incident cases and deaths, respectively, for the Socioeconomic Status theme. The trends were similar to those for overall SVI. Incident cases were higher for the least vulnerable quartile from March 15 through April 3, at which point the RRs became significantly >1.00. The RRs achieved a maximum of 2.80 (95% PI: 2.65, 2.94) on June 19 before a plateau in July. Starting in August, the RRs declined steadily as the per capita cases for the lower quartile began to catch up to the upper quartile. As with overall SVI, we observed a second crossover event on September 22 when the most vulnerable counties again experienced lower incidence rates compare to the least vulnerable counties. In fact, on November 9, the most vulnerable counties had 45% lower risk of infection on average compared to the least vulnerable counties (RR = 0.55, 95% PI: 0.51, 0.60). The trend reversed for a final time on December 7, and the RRs remained above 1.00 through December 31. The death rate trend for the SES theme was similar to the death trend for overall SVI. The most vulnerable counties had lower death rates on average early in the pandemic, higher rates in the summer and early fall, and lower rates again as winter approached.

### SVI theme: Household composition

**Fig 5:**
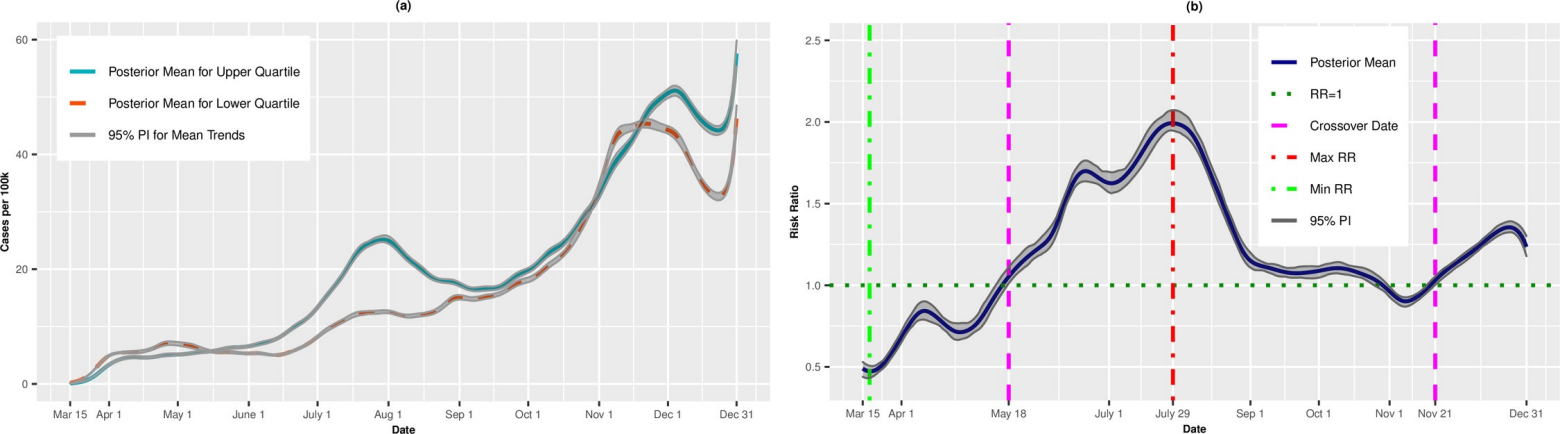
Incidence rates (A) and adjusted risk ratios (B) for upper (most vulnerable) versus lower (least vulnerable) quartiles of Household Composition.

**Fig 6:**
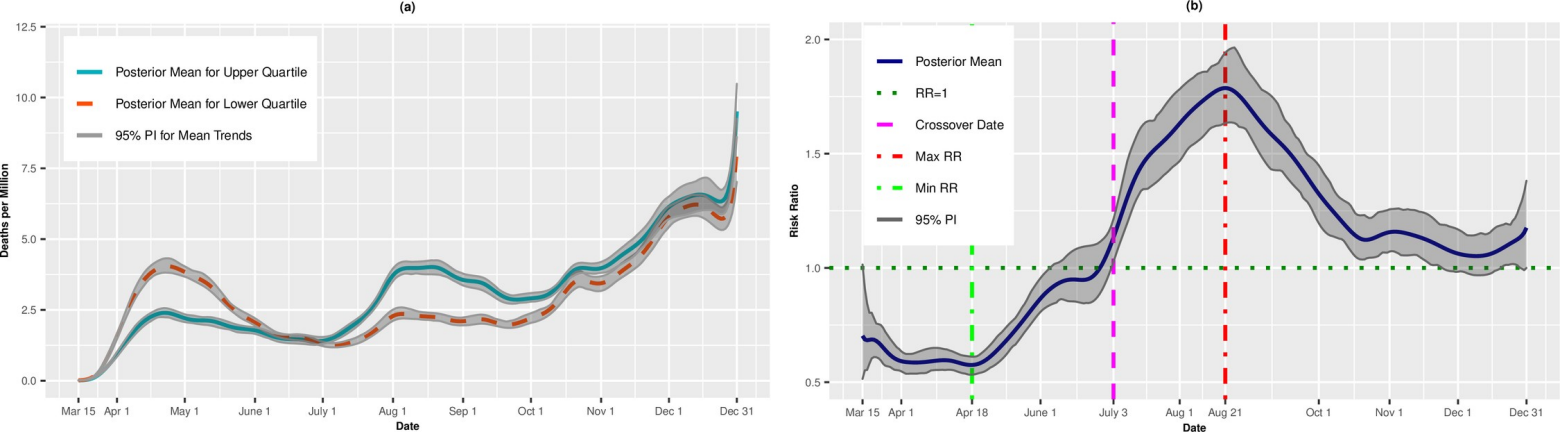
Death rates (A) and adjusted risk ratios (B) for upper (most vulnerable) versus lower (least vulnerable) quartiles of Household Composition. Maximum and minimum values, as well as the first and last crossover dates, are highlighted by vertical lines.

Figs 5A, 5B, 6A and 6B present the results for the Housing Composition theme. The crossover effect was significantly delayed for this theme, with the crossover dates occurring on May 18 for incident cases (Fig 5B) and on July 3 for deaths (Fig 6B). Thus, the pandemic appears to have disproportionately impacted the least vulnerable counties with respect to household composition for much of the early pandemic. However, these trends reversed by July. For incident cases, the daily RRs achieved a maximum of 1.99 (95% PI: 1.95, 2.07) on July 29 (Fig 5B) and then declined steadily. In late October and early November, there was another brief crossover period; however, on November 21, the trends reversed again, and the most vulnerable counties once more exhibited higher incidence than the least vulnerable counties. For deaths (Fig 6B), the maximum RR of 1.79 (95% PI: 1.63, 1.94) was achieved August 21 and, unlike incident cases, remained above 1.00 for the remainder of the study, although the 95% PIs overlapped 1.00 briefly in early December, indicating a null effect during this period.

### SVI theme: Race/ethnicity/language

**Fig 7:**
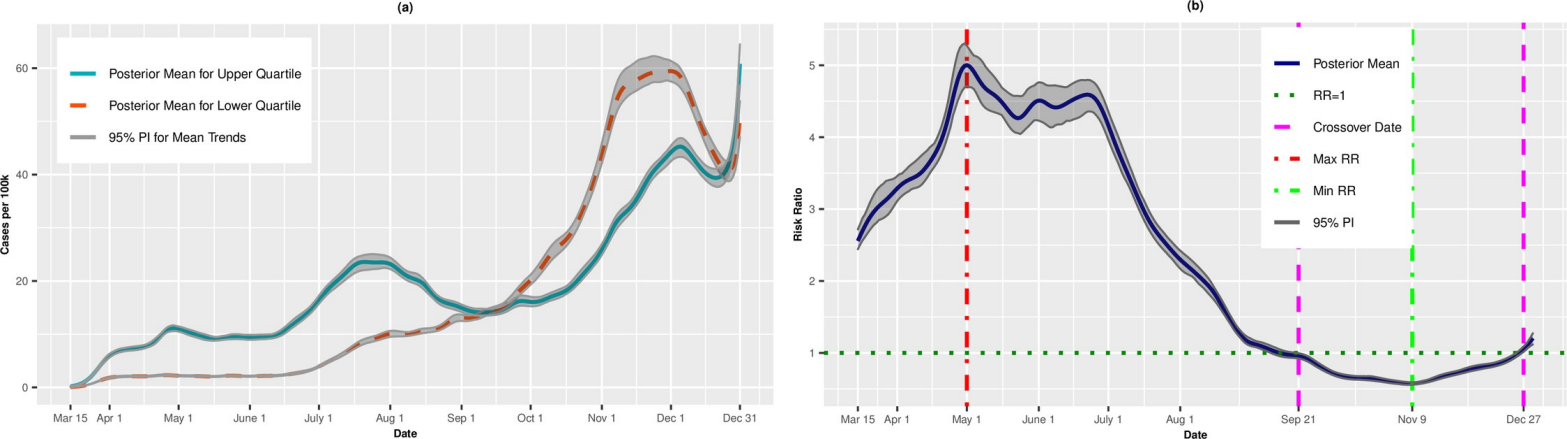
Incidence rates (A) and adjusted risk ratios (B) for upper (most vulnerable) versus lower (least vulnerable) quartiles of Race/Ethnicity/Language.

**Fig 8:**
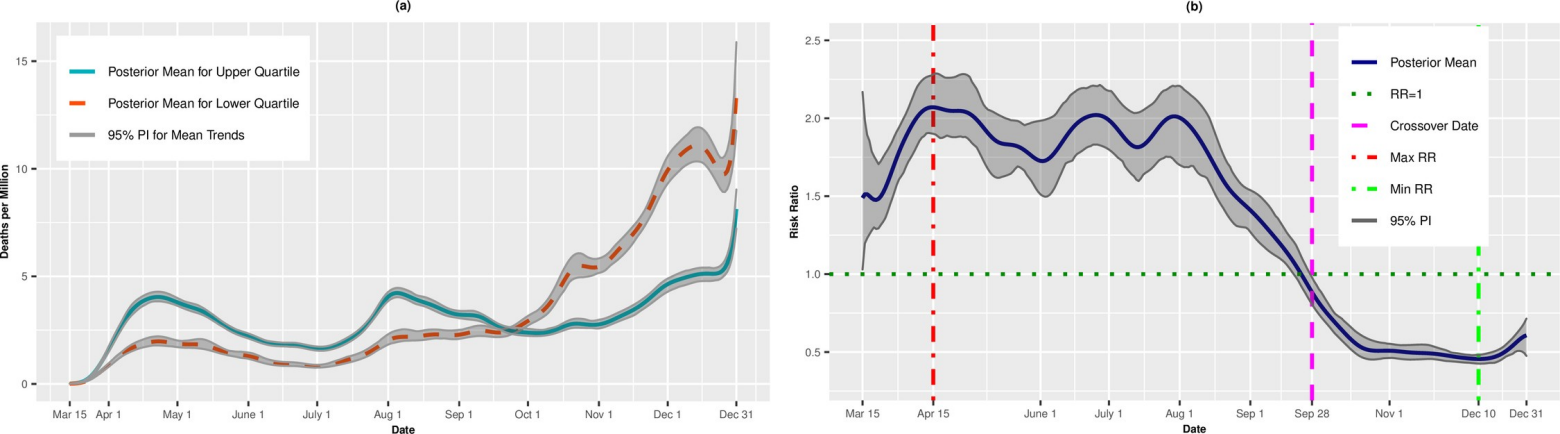
Death rates (A) and adjusted risk ratios (B) for upper (most vulnerable) versus lower (least vulnerable) quartiles of Race/Ethnicity/Language. Maximum and minimum values, as well as the first and last crossover dates, are highlighted by vertical lines.

Figs 7A, 7B, 8A and 8B present the results for the Race/Ethnicity/Language theme. Unlike the previous themes, the most vulnerable counties experienced higher incidence and death rates from the outset of the pandemic. In fact, the disparity between the most and least vulnerable counties was greatest for this theme, with a maximum incidence RR of 5.00 (95% PI: 4.71, 5.27) on May 1. For cases, the RRs declined steadily from late June into August, as the incidence for the lower quartile outpaced the upper quartile. On September 21, we observed the first crossover, and by November 9, the most vulnerable counties had 43% lower risk of infection (RR = 0.57, 95% PI: 0.56, 0.59). The death rates followed a similar pattern: the most vulnerable counties had higher death rates through mid-September when the trends reversed and the most vulnerable counties had lower death rates than the least vulnerable counties.

### SVI theme: Housing/transportation

**Fig 9:**
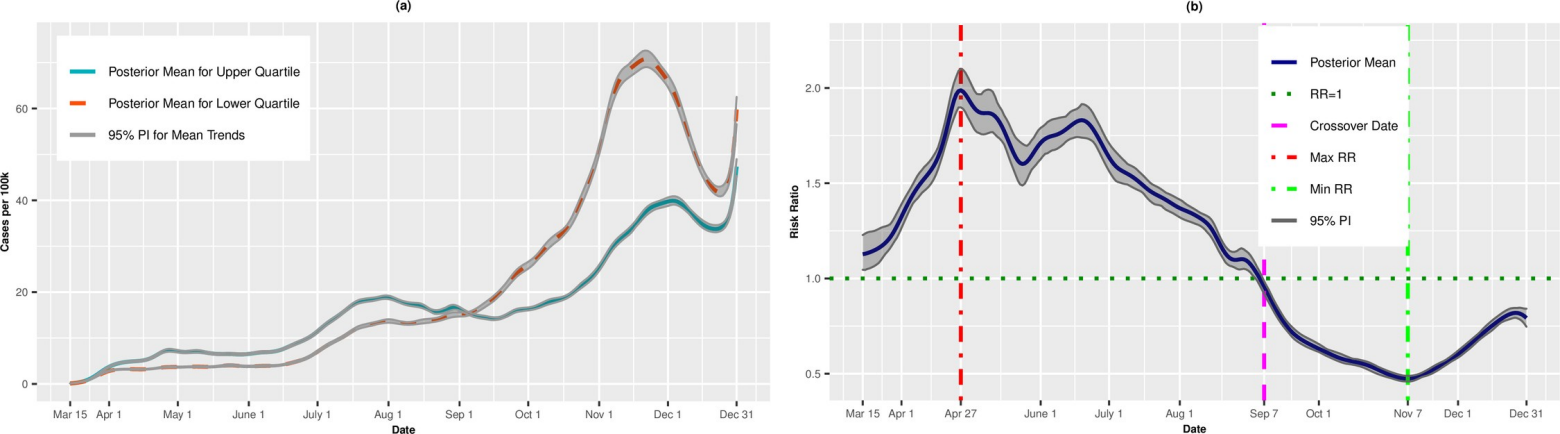
Incidence rates (A) and adjusted risk ratios (B) for upper (most vulnerable) versus lower (least vulnerable) quartiles of housing/transportation.

**Fig 10:**
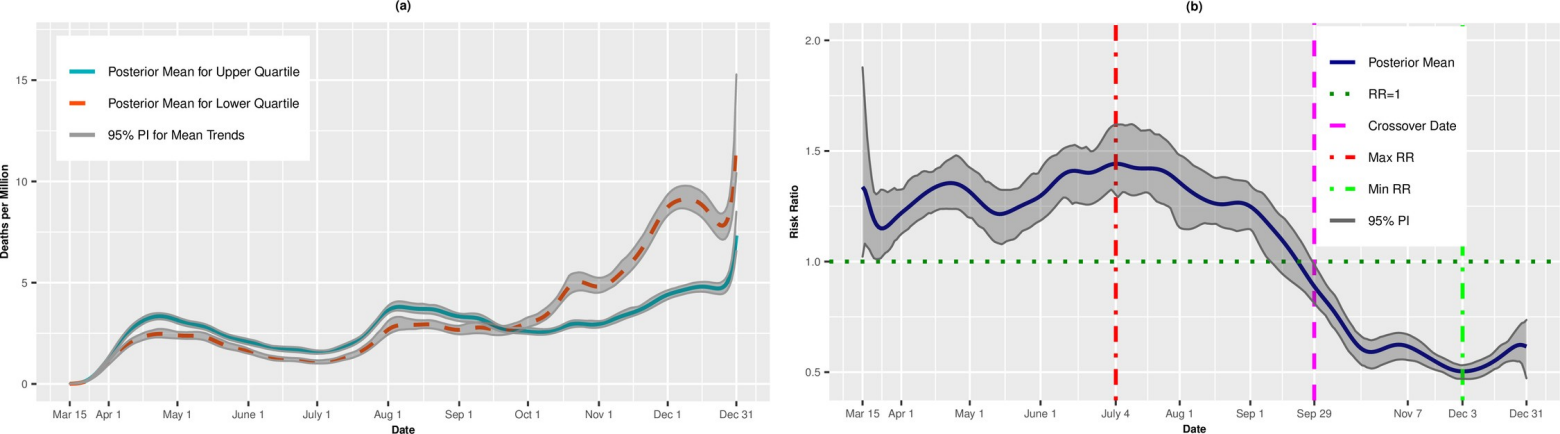
Death rates (A) and adjusted risk ratios (B) for upper (most vulnerable) versus lower (least vulnerable) quartiles of housing/transportation. Maximum and minimum values, as well as the first and last crossover dates, are highlighted by vertical lines.

Figs 9A, 9B, 10A and 10B present the results for the Housing/Transportation theme. The incident case RRs (Fig 9B) remained significantly positive from March 15 to September 7, with a maximum RR of 1.99 (95% PI: 1.90, 2.10) on April 27. However, from September 7 on, the most vulnerable counties had lower infection rates compared to the least vulnerable counties. The death rate RRs hovered between 1.25 to 1.5 through late August; on September 29, we observed a crossover effect whereby the most vulnerable counties experienced lower death rates.

There were also several significant associations among the adjustment variables. Table 3 presents the adjusted RRs and 95% PIs for the adjustment variables included in the overall SVI model. Percent rural, percent smoking, per capita number of primary care physicians, and average daily temperature were negatively associated with COVID-19 cases. Daily precipitation and population density were not significantly associated with cases. Similar results were observed for the death model, but here population density was positively associated with deaths, while precipitation had a modest negative association.

**Table 3:** Adjusted Risk Ratios (RRs) and 95% Posterior Intervals (PIs) for the adjustment variables included in the overall SVI models.

Model Outcome	Variable	Risk Ratio (95% PI)
Incident Cases	% female	1.04 (1.03, 1.06)
	% of county designated rural	0.86 (0.86, 0.87)
	% fair or poor health in county	1.23 (1.20, 1.25)
	% adult smokers in county	0.95 (0.93, 0.96)
	Average particulate matter (PM_2.5_) for county	1.13 (1.12, 1.15)
	Proportion tested in state	1.20 (1.20, 1.21)
	# primary care physicians per 100,000 in county	0.94 (0.93, 0.95)
	Average daily temperature (Fahrenheit)	0.92 (0.92, 0.93)
	Average daily precipitation (inches)	1.00 (1.00, 1.01)
	Population density	1.01 (0.99, 1.01)
Deaths	% female in county	1.10 (1.08, 1.12)
	% of county designated rural	0.91 (0.88, 0.93)
	% fair or poor health in county	1.49 (1.43, 1.53)
	% adult smokers in county	0.90 (0.87, 0.93)
	Average particulate matter (PM_2.5_) for county	1.14 (1.11, 1.17)
	Proportion tested in the state	1.41 (1.39, 1.43)
	# primary care physicians per 100,000 in county	0.93 (0.92, 0.96)
	Average daily temperature (Fahrenheit)	0.95 (0.92, 0.98)
	Average daily precipitation (inches)	0.96 (0.95, 0.98)
	Population density	1.02 (1.00, 1.03)

### Sensitivity analysis using Johns Hopkins data

Sensitivity analysis using the Johns Hopkins data produced similar results to those we observed using USAFacts data. S1A, S1B, S2A and S2B Figs present the incidence and death rate trends for overall SVI. For both cases and deaths, the trends were similar across the two data sources.

### Unadjusted sensitivity analysis

As a second sensitivity analyses, we ran corresponding unadjusted models for overall SVI. Results are presented in S3 and S4 Figs. The trends followed similar patterns to those in the adjusted analyses, although the RRs had a wider range in the unadjusted analysis. For example, the maximum RR for the unadjusted deaths was 3.27 (95% PI: 2.94, 3.55) on August 3 and 1.97 (95% PI: 1.76, 2.17) on August 14 in the adjusted analysis.

## Discussion

In this study examining COVID-19 incidence and death rates from March 15 to December 31, 2020, we found that cases were higher for the least vulnerable counties initially and toward the end of our study. However, from late March to late September, incident cases were substantially higher among the most vulnerable counties. Death rates followed a similar pattern. Thus, the RRs for both cases and deaths exhibited a sinusoidal pattern throughout the study. Three of the SVI theme (Race/Ethnicity/Language, Household Composition, and Socioeconomic Status) showed similar patterns to overall SVI. However, for Housing/Transportation, cases and deaths were higher for the most vulnerable counties from March through September. The trend reversed in October.

Our findings are generally consistent with the study by Khazanchi et al. examining data up to April 19, 2020, which found that counties in the upper quartile of overall SVI had higher incidence and death rates compared to those in the lower quartile [22]. As in that study, we found the strongest disparity for the Race/Ethnicity/Language theme in that the RRs achieved the largest values and were substantially higher for a large portion of the study period. However, Khazanchi et al. found no association with Household Composition, whereas we found that the most vulnerable had higher rates of cases and deaths from March to September. This may be due to the fact that the authors examined cumulative cases through April 19 only, whereas we examined daily incidence. However, this does not fully explain the difference, which could instead be due to our longitudinal approach, as it provided a comprehensive picture of the evolving relationship between SVI and COVID-19, rather than a momentary snapshot.

Our results may also shed light on inconsistent findings in two other studies. As in our study, Karaye et al. found that overall SVI and Race/Ethnicity/Language were associated with increased COVID-19 incidence through May 12, 2020 [20]. However, they found no association between Socioeconomic Status and incident cases, whereas Household Composition and Housing/Transportation had an inverse relationship. Our results place these findings in temporal context. In particular, we found multiple crossover effects for Household Composition, with differences varying over the observed across the study period. Nayak et al., meanwhile, found no association between overall SVI and cumulative COVID-19 incidence on April 4, 2020 [21]. According to our results, however, this was close to the first crossover date of March 19, when the most vulnerable counties had significantly higher rates if infection. Additionally, Nayak and colleagues found that the Race/Ethnicity/Language and Housing/Transportation themes were positively associated with incident cases, whereas we found multiple crossover effects. Again, these results highlight the need to consider both temporal and spatial variability when attempting to fully understand, in near-real time, the impact of the pandemic on populations with different vulnerability profiles.

Several covariates from our adjusted model were significantly associated COVID-19 cases and deaths. We found that rurality was associated with fewer cases and deaths, consistent with a prior study [22]. In contrast, percentage in poor or fair health was positively associated with both cases and deaths. This supports results from a recent study that found that patients with COVID-19 with cardiovascular disease, hypertension, diabetes mellitus, congestive heart failure, chronic kidney disease, and cancer had a higher risk of mortality, compared to patients with COVID-19 without these comorbidities [37]. Moreover, as in prior studies [20, 35], we found that average air quality, as measured by PM_2.5_, was positively associated with both cases and deaths. Increased state-level testing was also associated with higher rates of COVID-19 cases and deaths, likely due to heightened surveillance. Contrary to our expectation, we found a significant inverse association between percentage of adult smokers and COVID-19 cases and deaths. Our aggregated, county-level findings align with recent individual-level studies suggesting that nicotine may have a protective effect on COVID-19 [43, 44]. Finally, the number of primary care physicians per capita was associated with both lower incident cases and deaths.

More generally, our results suggest a dynamic impact of COVID-19 on socially vulnerable communities. Contrary to expectation, we found that COVID-19 disproportionately impacted less vulnerable counties early and later in the pandemic. The initial shift could reflect local and state policy decisions, such as early re-openings in states like Georgia with a high percentage of vulnerable counties [45, 46]. By late September, however, the least vulnerable counties began to keep pace with and eventually overturn the more vulnerable counties. This suggests that the impact of COVID-19 is not static, but can migrate from less vulnerable counties to more vulnerable ones and back again over time. These results highlight the need for communities, even less vulnerable ones, to continue to monitor the spread of the disease, maintain adequate health care resources, and remain vigilant in wearing masks and practicing social distancing.

There are, however, limitations to this analysis. First, we used county-level SVI data from 2018. It is possible that social vulnerability factors may have changed between 2018 and 2020, but we used the most recent SVI data available from CDC. Second, it was challenging to model deaths because many counties reported no deaths on any given day. Future studies could employ zero-inflated models to better account for this aspect of the data [47–49]. Future work could also examine temporal trends in locations of correctional facilities, long-term care facilities, nursing homes, Indian reservations and Tribal lands, and other places with high rates of infection [50–53]. Finally, we examined trends in the US only; future work might replicate our study in low- and middle-income countries or those with emerging outbreaks.

Examining the impact of COVID-19 on vulnerable communities in the US is important both during and after the pandemic [18, 54]. Overwhelming evidence suggests that social determinants of health and community contextual factors contribute to disparities in both COVID-19 incident cases and deaths [2, 3, 6, 55] and more recently in vaccinations [56]. It is therefore critically important to monitor and protect vulnerable populations as the pandemic continues to unfold.

## Supporting information

S1 Fig Incidence rates (A) and risk ratios (B) for overall SVI using Johns Hopkins data.
(TIF)

S2 Fig Death rates (A) and risk ratios (B) for overall SVI using Johns Hopkins data. Maximum and minimum values, as well as the first and last crossover dates, are highlighted by vertical lines.
(TIF)

S3 Fig Incidence rates (A) and risk ratios (B) for overall SVI in the unadjusted analysis.
(TIF)

S4 Fig Death rates (A) and risk ratios (B) for overall SVI in the unadjusted analysis. Maximum and minimum values, as well as the first and last crossover dates, are highlighted by vertical lines.
(TIF)

S1 Data (ZIP)
