# Supplementary figures and images for: Spatial and temporal trends in social vulnerability and COVID-19 incidence and death rates in the United States
Source: PLoS One. 2021 Mar 24;16(3):e0248702. doi: 10.1371/journal.pone.0248702 (PMC7990180; doi:10.1371/journal.pone.0248702)

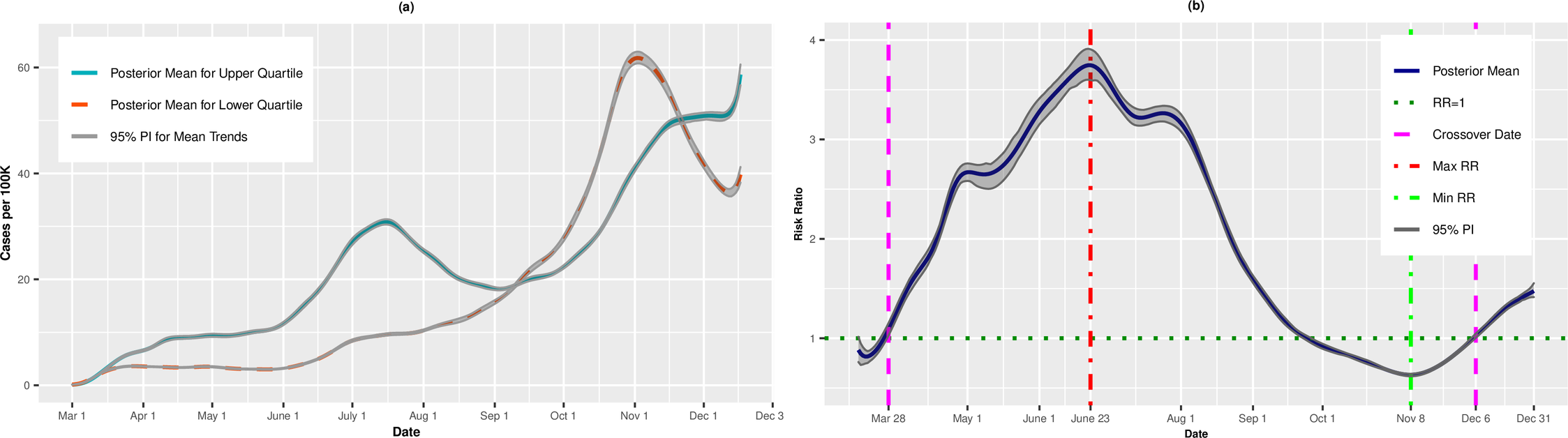

Supplement: S1 Fig — Incidence rates (A) and risk ratios (B) for overall SVI using Johns Hopkins data. (TIF) [file pone.0248702.s001.tif]

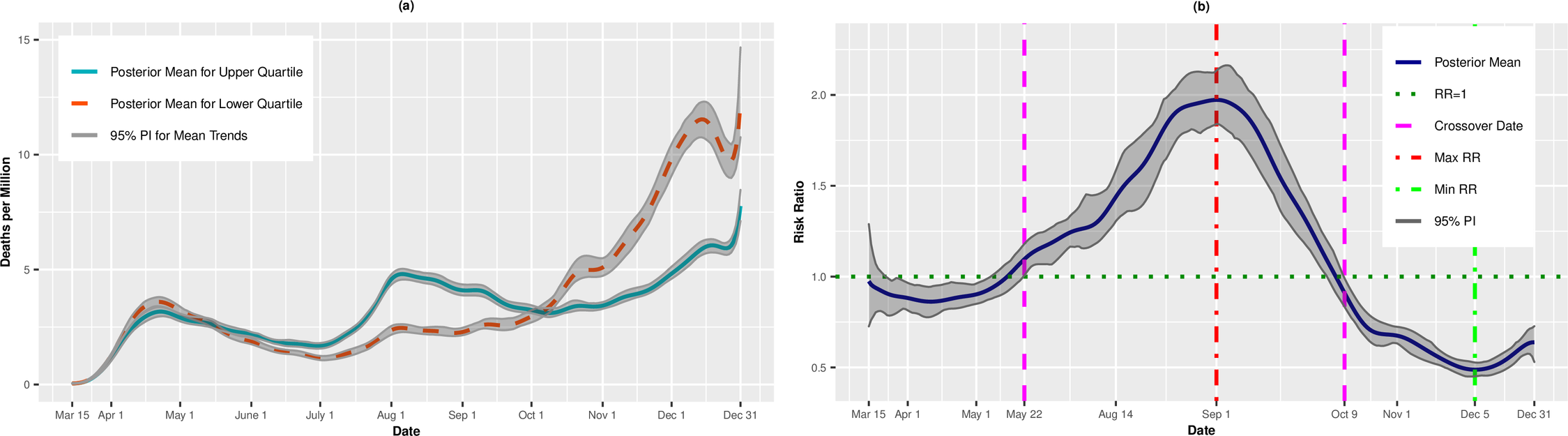

Supplement: S2 Fig — Death rates (A) and risk ratios (B) for overall SVI using Johns Hopkins data. Maximum and minimum values, as well as the first and last crossover dates, are highlighted by vertical lines. (TIF) [file pone.0248702.s002.tif]

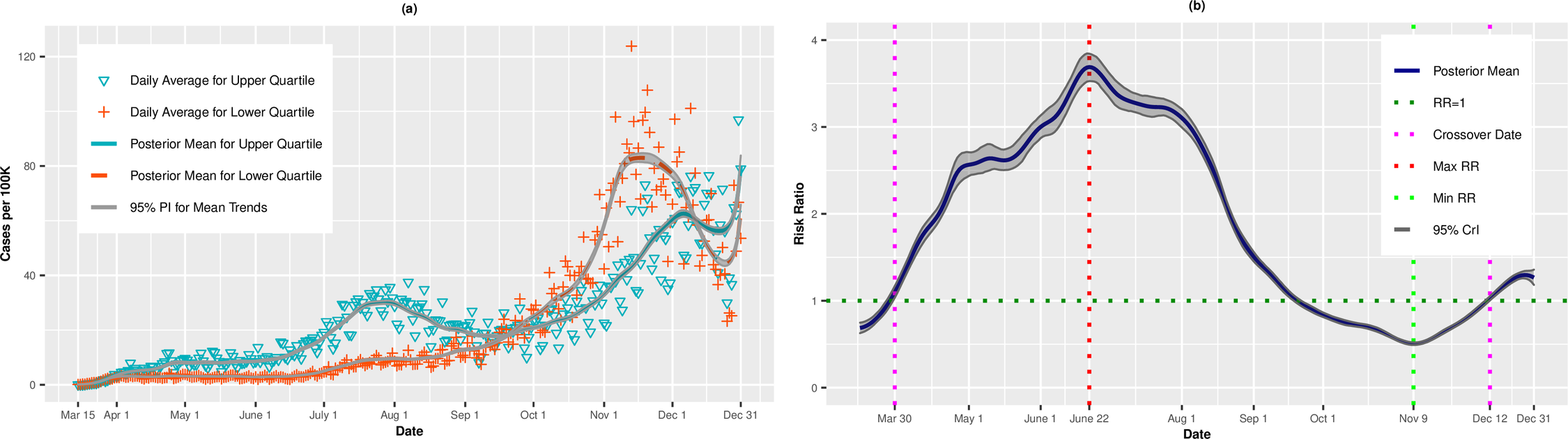

Supplement: S3 Fig — Incidence rates (A) and risk ratios (B) for overall SVI in the unadjusted analysis. (TIF) [file pone.0248702.s003.tif]

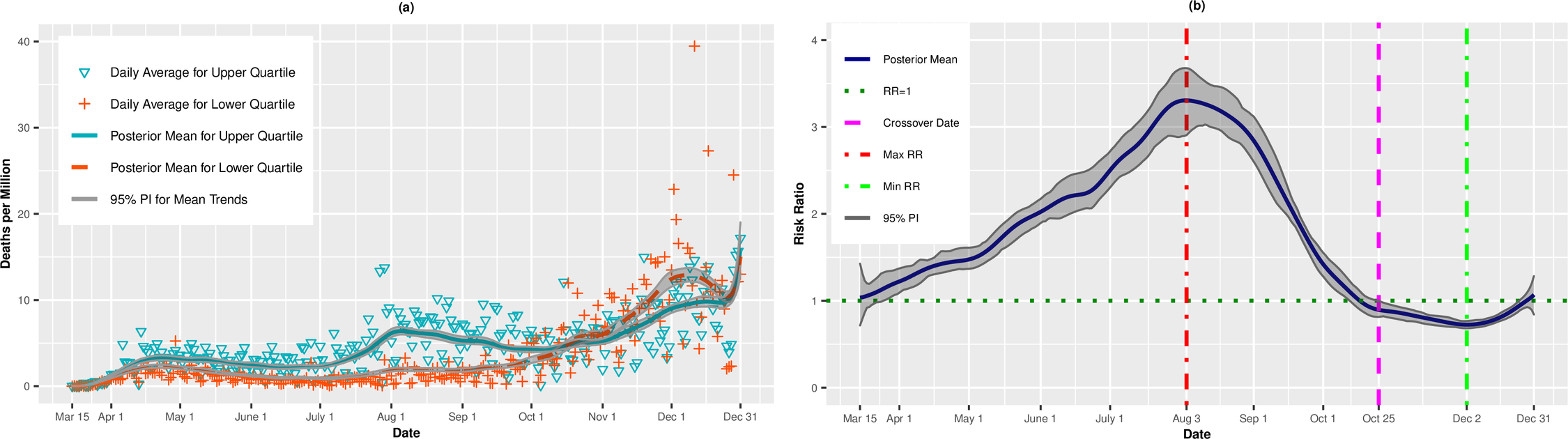

Supplement: S4 Fig — Death rates (A) and risk ratios (B) for overall SVI in the unadjusted analysis. Maximum and minimum values, as well as the first and last crossover dates, are highlighted by vertical lines. (TIF) [file pone.0248702.s004.tif]
